# A Bloodless Operation

**Published:** 1877-03

**Authors:** William Powell

**Affiliations:** Torrance, Miss.


TORRANCE, Miss., Feb. 16, 1877.

*Dr. E. S. Gaillard:*

A BLOODLESS OPERATION.—A negro woman requested me a short time since to get a piece of needle out of her arm, which she said had been accidentally lodged there about three years ago. I seated her near a table, applied a tourniquet buckled loosely about the middle of the arm, with the pad over the brachial artery; I then elevated the arm, with the hand higher than her head; an assistant pressed the pad, so as to stop the circulation in the artery; I then rubbed the hand and arm, forcing the blood from the limb through the veins to the body, then tightened the band of the tourniquet, so as to prevent all circulation of blood in the limb beyond; I then laid the hand on the table, made an incision through the skin about an inch long and extracted a piece of needle half an inch in length. The tissues were entirely blanched; I dressed the wound before removing the tourniquet, and did not see a particle of blood, the knife was not stained. This case demonstrates very fully the fact that the bloodless operation can be performed on the extremities without any new contrivance, the ordinary tourniquet being fully sufficient.—*Am. Med. Bi-Weekly.*

Respectfully, WILLIAM POWELL, M. D.

